# Catastrophic dynamics limit Atlantic cod recovery

**DOI:** 10.1098/rspb.2018.2877

**Published:** 2019-03-13

**Authors:** Camilla Sguotti, Saskia A. Otto, Romain Frelat, Tom J. Langbehn, Marie Plambech Ryberg, Martin Lindegren, Joël M. Durant, Nils Chr. Stenseth, Christian Möllmann

**Affiliations:** 1 Institute for Marine Ecosystem and Fisheries Science (IMF), Center for Earth System Research and Sustainability (CEN), University of Hamburg, 22767 Hamburg, Germany; 2 Department of Biological Sciences, University of Bergen, 5006 Bergen, Norway; 3 National Institute of Aquatic Resources, Technical University of Denmark (DTU Aqua), 2800 Kgs Lyngby, Denmark; 4 Department of Biosciences, Centre for Ecological and Evolutionary Synthesis (CEES), University of Oslo, 0316 Oslo, Norway

**Keywords:** catastrophe theory, stock collapse, Atlantic cod, stochastic cusp modelling, population recovery

## Abstract

Collapses and regime changes are pervasive in complex systems (such as marine ecosystems) governed by multiple stressors. The demise of Atlantic cod (*Gadus morhua*) stocks constitutes a text book example of the consequences of overexploiting marine living resources, yet the drivers of these nearly synchronous collapses are still debated. Moreover, it is still unclear why rebuilding of collapsed fish stocks such as cod is often slow or absent. Here, we apply the stochastic cusp model, based on catastrophe theory, and show that collapse and recovery of cod stocks are potentially driven by the specific interaction between exploitation pressure and environmental drivers. Our statistical modelling study demonstrates that for most of the cod stocks, ocean warming could induce a nonlinear discontinuous relationship between fishing pressure and stock size, which would explain hysteresis in their response to reduced exploitation pressure. Our study suggests further that a continuing increase in ocean temperatures will probably limit productivity and hence future fishing opportunities for most cod stocks of the Atlantic Ocean. Moreover, our study contributes to the ongoing discussion on the importance of climate and fishing effects on commercially exploited fish stocks, highlighting the importance of considering discontinuous dynamics in holistic ecosystem-based management approaches, particularly under climate change.

## 1. Introduction

Collapses and regime changes are pervasive in complex systems such as marine ecosystems [1–3] and can affect fish populations [4,5], trophic level communities [6–8] and entire large marine ecosystems [9–13]. Typically, such events are characterized by multiple external drivers that interact in causing abrupt changes and show hysteresis, a delayed or absent response to restoration and recovery efforts [3]. Anticipating and considering regime shifts is hence a crucial challenge for marine ecosystem-based management that has the goal of a sustainable exploitation of the oceans [14–16].

**Figure 1.**
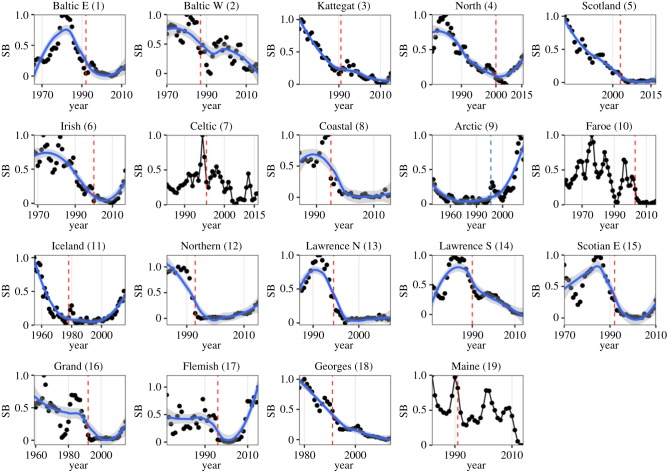
Spawner biomass (SB) trends and change points. Scaled SB (between 0 and 1, SB−min(SB)/max(SB)−min(SB)) time series of Atlantic cod stocks. Blue smoother lines indicate time trends and were fitted using generalized additive modelling (no smoother was fitted to stocks that mainly oscillate, in order to differentiate the two different stocks dynamics). Dotted vertical lines represent the major change points in the time series (red lines indicated negative, light-blue lines positive change points) derived by Bayesian change point and trend analysis (explained in the electronic supplementary material). Stock names and numbers according to the electronic supplementary material, table S1. (Online version in colour.)

The demise of Atlantic cod (*Gadus morhua*) stocks, which has been often linked to a combination of unsustainable fishing pressure and unfavourable climatic conditions, resulting in trophic cascades and feedback loops, constitutes a text book example of the consequences of overexploiting marine living resources (figure 1) [17–24]. Given the dire ecological and socio-economic consequences of these collapses [25], a wide range of management measures has been implemented in the attempt to promote the recovery of cod on both sides of the North Atlantic [26]. Unfortunately, most of these recovery measures have proved inefficient, indicating that cod recovery might be hindered by complex synergistic, antagonistic or additive interactions between multiple pressures [27,28]. We here analysed trends of 19 collapsed cod stocks (figure 1; electronic supplementary material, text and tables S1 and S2) finding only two stocks fully recovered, and six in the process of recovering (electronic supplementary material text, and figures S1 and S2). Eleven cod stocks can still be considered depleted, causing a great deal of controversy regarding the underlying processes of failed recovery both in the scientific literature [20] but also in popular media [26]. Here, we address the question of how fishing pressure and climatic changes (represented by sea surface temperature (SST)) interact to cause patterns of collapse and recovery of Atlantic cod stocks, applying an approach based on catastrophe theory. The approach allows us to detect how multiple drivers can interact to cause discontinuous dynamics and we suggest that its application can facilitate the understanding of the recovery potential of depleted living marine resources in general. Understanding the recovery potential of Atlantic cod is especially important because the species is not only a fundamental component of marine ecosystems, but also one of the most requested living marine resource species on the international market [29,30].

Catastrophe theory is a branch of bifurcation theory in the field of nonlinear dynamical systems that studies and classifies phenomena characterized by sudden shifts in behaviour derived from small changes in external conditions [31]. The theory, developed by the French mathematician René Thom in the 1960s [32] and popularized by Christopher Zeeman in the 1970s [33], became somewhat an intellectual fad [31]. Catastrophe theory was believed to be applicable to every branch of science and hence was quickly embraced by scientists in diverse fields. Examples of applications in marine ecology and resource management questions included models of fishery dynamics [34–36] and predator–prey interactions of Great Lakes trout [37]; but as quickly as it became popular, the theory started to be heavily criticized [38–41] which resulted in a major debate on its usefulness and potential misuse, in particular owing to its original deterministic framework (responses appeared in *Science* and *Nature* in 1977 [42–44]). This debate gradually undermined the support for using catastrophe theory and led to a widespread dismissal until the 2000s [31].

**Figure 2.**
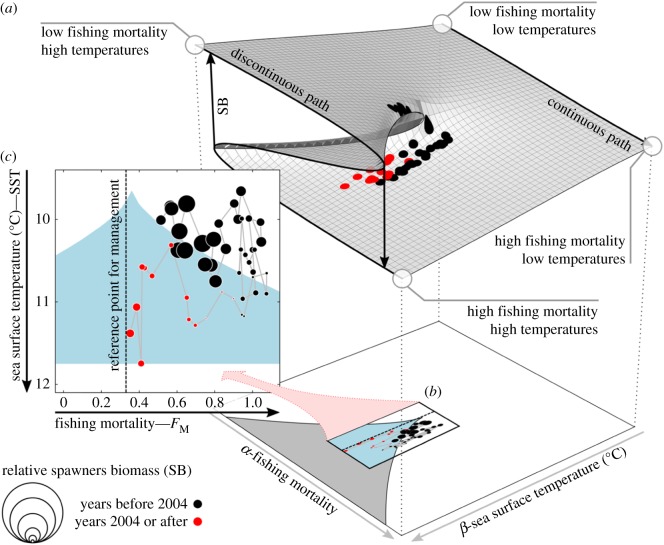
The stochastic cusp model—from three-dimensional to two-dimensional representation. (*a*) The typical three-dimensional representation of the cusp model where North Atlantic cod SB dynamics depend on two controlling variables *α* (fishing mortality—*F*_M_) set by fisheries management and *β* (sea surface temperature—SST) controlling whether SB follows a continuous or discontinuous path. (*b,c*) Two-dimensional projection of the plane. The bifurcation area under the folded three-dimensional phase plane is shaded in grey and light blue (representing where the data of this stock can be found in the plane). Filled dots in (*b*) and (*c*) represent SB with the radius scaling relative to stock size. The red dots are highlighted in order to show the last *ca* 10 years of the time series. (*c*) The vertical dotted line represents the present management target, in this case *F*_MSY_, which can be found in the electronic supplementary material, table S3. Note that the *y*-axis is reversed with temperature increasing downwards.

Standard catastrophe theory differentiates seven elementary catastrophes (canonical forms) that can describe systems characterized by abrupt shifts with up to six dimensions in control and state variables [32]. Most of the applications of catastrophe theory use the two simplest forms, the fold and the cusp. The fold catastrophe describes sudden changes of a dynamic system in response to a single pressure variable and has been widely used in ecology to discuss concepts such as resilience and hysteresis [45,46]. The cusp catastrophe, in contrast with the fold, considers a three-dimensional system (figure 2) where a second external variable acts as splitting factor that can modify the system's response to the principal external driver from linear and continuous to nonlinear and discontinuous. The cusp catastrophe is hence an ideal model to evaluate the effect of two interacting drivers such as fishing pressure and environmental forces on ecological systems, a potential that has not been sufficiently exploited yet (but see [47]). A major criticism of early studies applying the deterministic catastrophe theory was their descriptive nature owing to the lack of a stochastic framework [48]. The recent development of such a framework to cusp modelling has revived interest in the concept with an increasing number of publications in disciplines such as economy [49], sociology and behavioural science [48].

Although the cusp catastrophe may be ideal for explaining abrupt changes in ecological systems, which are often owing to the interaction of multiple external drivers [2,3], the model is still rarely considered [47]. Here, we applied the stochastic cusp model to 19 Atlantic cod stocks to understand: (i) whether their dynamics follow a continuous or discontinuous path, (ii) how fishing and environmental drivers interact in inducing discontinuous dynamics, and (iii) how discontinuous dynamics affect the recovery potential of Atlantic cod stocks.

## 2. Material and methods

### (a) Data

In order to represent the population dynamics of 19 Atlantic cod stocks, we collected time series of comparable spawner biomass (SB) (i.e. biomass of mature fish in tonnes) and fishing mortality estimates derived from stock assessments. Data were provided by the International Council for the Exploration of the Sea (ICES), the National Oceanic and Atmospheric Administration of the USA (NOAA), the Northwest Atlantic Fisheries Organization (NAFO) and the Department of Fisheries and Ocean in Canada (DFO) (electronic supplementary material, tables S1 and S2). A few recent stock assessments (i.e. the Kattegat, the western Baltic and the Norwegian coastal cod) comprised only reduced assessment periods. Where possible, we extended the SB and fishing mortality time series by combining them with comparable estimates from previous assessments after performing consistency checks (electronic supplementary material, figure S5).

To represent changes in environmental conditions experienced by each stock, we collected time series of SST (in °C). Although SST does not fully reflect the thermal habitat of cod, a predominately demersal (bottom-dwelling) species, SST has previously been shown to be a strong predictor influencing cod stock dynamics, including reproduction and growth [50,51]. SST data were collated from the NOAA Extended Reconstructed Sea Surface Temperature dataset (ERSST, www.ncdc.noaa.gov) v. 4. The dataset represents a reconstruction of SST from 1854 to the present and represents monthly anomalies computed with respect to the period 1971–2000, resolved in a 2° × 2° grid of spatial coverage. For every stock, we calculated the mean annual SST values averaged over the management area (electronic supplementary material, table S2). As the eastern Baltic cod stock is not strongly influenced by temperature but rather affected by oxygen, the annual extents of anoxic areas (in km^2^) [52] were used as environmental covariate for this particular stock.

### (b) Stochastic cusp modelling

We tested if a statistical approach to catastrophe theory could explain collapse and recovery patterns of Atlantic cod stocks. Catastrophe theory provides a mathematical framework to model both continuous and discontinuous changes in a system's dynamics [32,47,49]. In particular, it is effective in describing abrupt changes in the state variable as a result of small and continuous changes in control variables [32,48]. The cusp catastrophe describes sudden and discontinuous transitions in the equilibrium state of a state variable *Z_t_* depending on two control parameters *α* and *β*. The canonical form of its potential function is

2.1 $$-V(Z_{t};\alpha ,\beta )=-\frac{1}{4}Z_{t}^{4}+\frac{1}{2}\beta Z_{t}^{2}+\alpha Z_{t}.$$

In order to be applicable to empirical data, which often present stochasticity, equation (2.1) was reformulated as a stochastic differential equation by adding a (white noise) Wiener process with variance *σ*^2^:

2.2 $$dZ_{t}=(-Z_{t}^{3}+\beta Z_{t}+\alpha )dt+\sigma_{z}dW_{t},$$

where the first part of the equation is the drift term, *σ*_z_ is the diffusion parameter and *W_t_* represents the Wiener process.

The parameters *α* and *β* were estimated as linear functions of exogenous variables, while the dependent canonical state variable (*Z_t_*) is a linear function of one or more observable dependent state variables using a likelihood approach (below equations) [48,49]:

2.3a $$\alpha =\alpha_{0}+\alpha_{1}F_{M},$$

2.3b $$\beta =\beta_{0}+\beta_{1}\mathrm{SST},$$

2.3c $$\mathrm{and}\;Z=w_{0}+w_{1}\mathrm{SB},$$

where $\alpha_{0},\beta_{0}\;\mathrm{and}\;\;w_{0}$ are the intercepts and $\alpha_{1},\beta_{1}\;\mathrm{and}\;\;w_{1}$ are the slopes of the models.

In our study, we fitted the state variable (*Z*) as a linear function of cod SB (figure 2, equation (2.3*c*)). The control parameters *α* and *β* are called asymmetry and bifurcation variables, respectively [48,49]. The asymmetry variable (*α*) regulates the dimension of *Z_t_* and was fitted as a linear function of fishing mortality (*F*_M_), set by fisheries management and commonly assumed to be linearly related to population size [53] (figure 2, equation (2.3*a*)). The bifurcation variable (the splitting factor, *β*) determines whether the state variable follows a continuous or discontinuous path, and in our case, was fitted as a linear function of SST (figure 2, equation (2.3*b*)). We used SST as an accepted proxy for environmental conditions affecting biological processes such as recruitment [54,55] and growth in Atlantic cod [56], and as an indicator for climate change effects [51,57]. An exploratory analysis accounting for lagged effects of SST on SB did not result in significantly different outcomes and hence we here present the non-lagged models only (electronic supplementary material, table S6). The obtained parameters were then fitted into equation (2.1) to ultimately understand whether cod stocks follow continuous or discontinuous dynamics.

Equilibria of the system corresponding to the solution of the cubic equation are as follows:

2.4 $$-\frac{\partial V(Z;\alpha ,\beta )}{\partial z}=-Z^{3}+\beta Z+\alpha =0.$$

From equation (2.3), a Cardan's discriminant (*δ*, equation (2.5)) is derived [49], that allows us to distinguish if the system is in a state with only one (*δ* > 0) or three equilibria (*δ* < 0):

2.5 $$\delta =27\alpha^{2}-4\beta^{3}.$$

Our cusp modelling approach can be visualized as a three-dimensional landscape where the trajectory of cod stock size in response to changing fishing mortality can be continuous (i.e. linear, with one stable equilibrium) or discontinuous (i.e. folded, with two stable equilibria and one unstable equilibrium) depending on SST (based on the results of equation (2.5)). As an example, figure 2 presents the collapse of North Sea cod, indicated by the drop in SB from the upper to the lower shield of the phase plane owing to high fishing pressure (figure 2*a*). The stock was modelled to be below the folded area and thus to move between two stable states. The bifurcation set is the area under the fold where one unstable state is present, and thus where the dynamics of the stock can be unpredictable (i.e. *δ* = 0). After the collapse, and with increasing temperatures, SB values progressively move below the discontinuous fold into the bifurcation set. Thus, critical thresholds are readily breached by relatively minor changes in fishing mortality, causing stocks to potentially fluctuate between the two alternative states along the discontinuous path. Stocks following a discontinuous path, and thus staying close to the bifurcation area, are prone to tipping points.

A projection on the two-dimensional plane allows one to follow the stock dynamics of North Sea cod and understand why its recovery may be limited (figure 2*b*,*c*). Stock size decreased in response to increasing fishing mortality, moving in and out the bifurcation set (indicated in light blue). Eventually, the stock collapsed to a very low biomass state and remained in the unpredictable area of the cusp. During the last 10 years of the time series (indicated in red in figure 2), exploitation pressure of North Sea cod has been drastically reduced; however, SB levels remained significantly lower compared to the beginning of the study period, when fishing mortality was similar. This hysteresis in response to decreased exploitation pressure is related to an increase in SST that is detrimental for North Sea cod [55]. Therefore, the cusp model can also explain hysteresis in the recovery of the state variable (i.e. SB of Atlantic cod stocks). Recovery of a collapsed fish population can then either occur when SST changes in a way that fishing mortality again has a linear effect on SB, or, within the bifurcation area, when chance events, e.g. high reproductive success, occur.

### (c) Model validation and comparison

We applied the stochastic cusp model to investigate how the interaction of fishing pressure and environmental conditions affects patterns of collapse and recovery of Atlantic cod stocks. We carefully validated the fitted cusp models following criteria proposed by Grasman *et al*. [48]. We specifically explored the significance of SB in the model of the canonical state variable *Z* (based on equation (2.3*c*)) (table 1; electronic supplementary material, table S5), the existence of bimodality of the state variable in the bifurcation area (electronic supplementary material, figure S3) and the percentage of observations in the bifurcation area (greater than 10% being the benchmark) (electronic supplementary material, table S4). To assess the goodness of fit of the model, we used the Cobb's pseudo-*R*^2^ (table 1) [48].

**Table 1:** *.* Results of the valid stochastic cusp models. (Results of valid (see the electronic supplementary material, table S4) *cusp models* for Atlantic cod stocks (stock numbers according to the electronic supplementary material, table S1 are indicated in parentheses). Reported are estimated model parameters (with standard errors) *α*_0_/*α*_1_ (for fishing mortality—*F*_M_), *β*_0_,/*β*_1_ (for sea surface temperature—SST; except for Baltic E where the extent of anoxic area were used as a predictor) and for *w*_0,_/*w*_1_ (SB, as the state variable). Asterisks indicate the significance level of the estimated parameters (*p < 0.05, **p<0.005, ***p<0.0005). Furthermore, the *R*^2^ (Cobb's pseudo-*R*^2^) indicates the quality of the cusp model fit and the AIC_c_ is given for comparison of the cusp and the alternative linear and logistic models.)

stock	*α*_0_	*α*_1_	*β*_0_	*β*_1_	*w*_0_	*w*_1_	*R*^2^	AICc (cusp)	AIC_c_ (linear)	AIC_c_ (logistic)
Baltic E (1)	0.12 (0.47)	−0.83 (0.58)	0.77 (0.91)	3.76 × 10^−2^(1.83 × 10^−2^)*	−2.6 (0.16)***	6.75 × 10^−6^ (5.1 × 10^−7^)***	0.77	107	1271	1252
Baltic W (2)	0.19 (1.22)	−0.17 (1.10)	4.59 (3.49)	−0.37 (0.366)	−2.50 (0.37)**	8.41 × 10^−5^ (1 × 10^−5^)***	0.69	137	1016	1015
Kattegat (3)	0.83 (0.59)	−1.38 (−0.62)*	−13.40 (4.39)**	1.53 (0.43)***	−2.41 (0.16)***	1.28 × 10^−4^ (1.04 × 10^−5^)***	0.75	93	923	921
north (4)	0.65 (0.47)	−1.81 (0.76)*	−18.78 (5)***	1.95 (0.46)***	−3.06 (0.22)***	1.65 × 10^−5^ (1.69 × 10^−6^)***	0.38	124	1335	1327
Scotland (5)	1.77 (1.90)	−3.04 (2.31)	−54.01 (10.6)***	5.26 (1)***	−2.54 (0.18)***	9.27 × 10^−5^ (1.07 × 10^−5^)***	0.64	67	751	745
Irish (6)	−0.08 (0.32)	−0.46 (0.35)	−42.79 (9.6)***	3.94 (0.83)***	−2.4 (0.16)***	1.993 × 10^−4^ (2.1 × 10^−5^)***	0.59	108	975	971
coastal (8)	−0.68 (0.43)	−0.71 (1.24)	−10.97 (3.5)***	4.49 (1.16)***	−2.57 (0.178)***	2.07 × 10^−5^ (1.82 × 10^6^)***	0.77	59	810	797
Arctic (9)	2.42 (0.75)**	−9.86 (2.67)***	20.88 (3.70)***	−5.51 (0.15)***	−3.17 (1.1 × 10^−7^)***	1.731 × 10^−6^ (1.1)	0.78	53	1978	NA
Iceland (11)	4.65 (1.17)***	−14.91 (3.75)***	3.96 (4.17)	−0.35 (0.15)*	−3.17 (3.5 × 10^−7^)***	5.465 × 10^−6^ (0.596)	0.77	70	1649	1637
northern (12)	−1.60 (0.63)*	9.03 (3.48)**	4.019 (3.05)	0.03 (0.15)	−2.48 (0.55)***	5.21 × 10^−6^ (3.09 × 10^−7^)***	0.94	20	922	899
Lawrence N (13)	−0.34 (0.17)	0.07 (0.30)	−4.38 (2.32)	1.43 (0.43)**	−2.53 (0.14)***	2.41 × 10^−5^ (1.47 × 10^−6^)***	0.85	72	1024	1021
Lawrence S (14)	−0.66 (0.22)**	1.55 (0.97)	−14.72 (3.33)***	2.53 (0.51)***	−3.01 (0.20)***	1.35 × 10^−5^ (1.04 × 10^−6^)***	0.65	92	1128	1106
Scotian E (15)	−0.49 (0.19)**	0.69 (0.37)	−22.28 (9.98)*	1.63 (0.66)*	−2.14 (0.16)***	2.67 × 10^−5^ (2.095 × 10^−6^)***	0.80	96	1003	974
Grand (16)	−0.88 (0.28)**	1.43 (0.52)**	−5.4 (5.39)	0.61 (0.47)	−1.88 (0.15)***	3.46 × 10^−5^ (2.95 × 10^−6^)***	0.62	138	1316	1302
Flemish (17)	−0.52 (0.23)*	0.59 (0.48)	−31.38 (7.35)*	2.19 (0.50)*	−2.13 (0.17)***	1.12 × 10^−4^ (1.07 × 10^−5^)***	0.69	108	912	923
Georges (18)	2.01 (0.81)*	−3.46 (1.25)**	−15.12 (8.74)	1.23 (0.62)*	−2.32 (0.17)**	4.42 × 10^−5^ (3.58 × 10^−6^)***	0.76	75	837	830

The fitted cusp models were further compared to the more parsimonious alternative linear and logistic regression models [48]. These models are often used to confront linear and continuous dynamics with the discontinuous regime shift case represented by the cusp model [45,58].

The linear model is here a simple multiple linear regression:

2.6 $$Z=g_{0}+g_{1}\times \mathrm{FM}+g_{2}\times \mathrm{SST}+\epsilon ,$$

where *g*_0_ represents the intercept of the model and *g*_1_ and *g*_2_ the coefficients of the two control variables, while ϵ is the normally distributed random error (mean = 0, variance = *σ*^2^).

In contrast with the linear model, abrupt changes of the state variable depending on the two control parameters can be represented by the logistic model

2.7 $$Z=\frac{1}{1+e^{\left(-\alpha /\beta^{2}\right)}}+\epsilon ,$$

where Z, *α* and *β* are canonical variables of observed state and control variables defined in equation 2.3(*a*–*c*), and ϵ is the zero mean random disturbances.

However, the logistic model does not represent the interaction of external drivers, cannot model critical points and thus does not reveal discontinuous dynamics of the state variable [48]. Hence, the comparison between the stochastic cusp model and the alternative linear and the logistic models allows us to understand if cod stock dynamics are better described by true nonlinear discontinuous dynamics induced by the interaction of external drivers, or by linear continuous dynamics (equations (2.6) and (2.7)) that ignore such interactions (see [45]).

All models were fitted using a maximum-likelihood approach under the assumption of normal errors and compared using Akaike's information criterion (AIC) [48]. All analyses were conducted in the statistical programming environment R [59] with RStudio (v. 3.3.1) [60] using the R package cusp [61], in particular the function ‘cusp’.

## 3. Results and discussion

**Figure 3.**
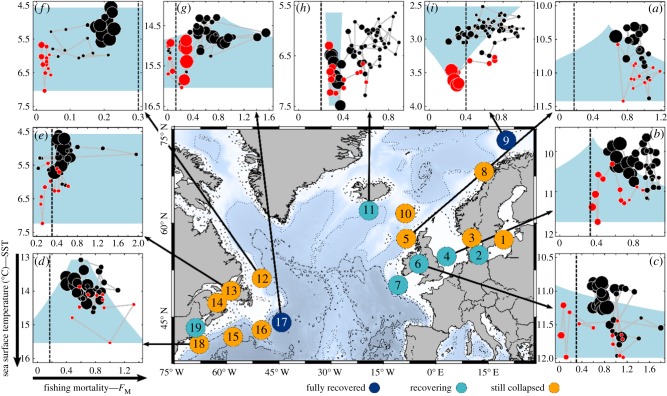
Two-dimensional bifurcation plots of the stochastic cusp model. Map indicating 19 North Atlantic cod stocks (number according to the electronic supplementary material, table S1) and their recovery status. Panels show cusp model results for nine stocks ((*a*), West of Scotland; (*b*), North Sea; (*c*), Irish Sea; (*d*), Georges Bank; (*e*), northern Lawrence; (*f*), northern cod; (*g*), Flemish Cap; (*h*), Iceland; (*i*), north east Arctic); other stocks, see Extended Data. Dots represent SB scaled to stock size; years greater than 2004 in red. The bifurcation area is shaded in blue and vertical dashed lines indicate stock specific management reference points of fishing mortality (*F*_M_) (electronic supplementary material, table S3). Note that the *y*-axis is reversed with temperature increasing downwards. (Online version in colour.)

We applied stochastic cusp modelling to 19 cod stocks from both sides of the North Atlantic (figure 3). To our knowledge, our study is one of the few to apply this methodology to empirical data from an ecological system [62]. The model evaluation exploring the percentage and bimodality of the observations in the bifurcation area [48] revealed 16 out of the 19 cusp models to be valid (electronic supplementary material, tables S4 and S5 and figure S3). Moreover, generally, the cusp model provided better statistical fits to the data than alternative linear and logistic models for all Atlantic cod stocks (table 1). Importantly, the model results indicate that in 13 out of the 16 valid cusp models, SST was a significant predictor of cod SB dynamics. Additionally, fishing mortality was a significant predictor in five cases, and the only significant predictor in two cases (table 1). These results show that Atlantic cod stock dynamics are well described by discontinuous, catastrophic behaviours determined by fishing pressure and temperature, as also suggested by previous studies [22,24,45,63].

Eastern Atlantic cod stocks best demonstrate this catastrophic behaviour, as also shown for North Sea cod (figure 1). Warming moves the trajectory of the stock towards the discontinuous region (i.e. down the *y*-axis) and into the bifurcation set (figure 3*a–c*; electronic supplementary material, figure S4). Here, even minute changes of fishing mortality are sufficient to drive the stock to collapse, because the stock is on the verge of the fold. Similar dynamics are observed for western Atlantic stocks (figure 3*d–f*; electronic supplementary material, figure S4) where most of the SB observations are found within the bifurcation set. The bifurcation set is the area of the fold where just one unstable equilibrium exists [45,64], indicating unstable dynamics [65]. Therefore, western stocks seem to be more vulnerable to catastrophic changes, probably owing to differences in life-history traits, local oceanic conditions and exploitation history [20,66,67]. Apart from stocks in the Baltic Sea and at the Norwegian coast (electronic supplementary material, figure S4), eastern Atlantic cod stocks show higher instabilities owing to the temperature increase in recent years (highlighted in red in figure 3). This recently very low resilience of the eastern cod stocks advocates for the application of more precautionary management measures, i.e. harvest control rules with lower fishing opportunities to counteract the high uncertainties in cod stock dynamics [15,58,68].

The stochastic cusp model allowed us to better understand the interacting effects of fishing pressure and ocean warming on Atlantic cod dynamics. All stocks, except the highly vulnerable Norwegian coastal and northern cod (where most of the data points are in the bifurcation set), collapsed at a fishing mortality well above sustainable levels, i.e. *F*_MSY_ (figure 3; electronic supplementary material, figure S4 and table S3), indicating the paramount importance of fishing pressure in regulating cod stock size [69]. Specifically, fishing below or around *F*_MSY_ would have maintained larger stock sizes and reduced the vulnerability of these stocks to SST changes, as indicated by stocks falling outside the bifurcation set (see especially North Sea and West of Scotland cod; figure 3*a,b*). Recent management efforts have often reduced fishing mortality to near or far below *F*_MSY_ such as in north and Irish Sea cod (figure 3*a,c*). While these stocks can be considered recovering (electronic supplementary material, figures S1 and S2), SB remains in most cases far below historical levels at similar or higher exploitation pressure. This hysteresis towards recovery is particularly evident in the western Atlantic stocks (e.g. southern Gulf of St Lawrence) (electronic supplementary material, figure S4), which after more than 20 years still reside in a depleted state. According to our cusp models, such hysteresis is owing to temperature changes that modified the relationship between fishing mortality and SB from linear to discontinuous, causing limited recovery of the biomass even after the reduction in fishing mortality [45].

Our results confirm the importance of fishing pressure and oceanic temperature conditions and, for the first time to our knowledge, identify their interacting effects on fish populations [20,21,70]. In particular, the hysteresis effect is in most of the cod stocks caused by an increase in SST. SST can have an effect directly on cod stocks through recruitment [54,57], growth [56] and mortality as well as indirectly through predator–prey switches or habitat degradation [71,72]. Moreover, hysteresis could also be because of trophic cascading observed in many cod-dominated systems, where the decrease in cod has induced increases of forage fishes hindering the comeback of cod [22–24,29,73]. Thus, the presence of hysteresis implies that recovery may only occur after a prolonged period of very low fishing mortality, or may not happen at all. Sudden increases in SB are theoretically possible as demonstrated by the recently recovered Flemish Cap cod (figure 3*g*). Still, a long-term reduction in fishing mortality is necessary to increase survival and year-class strength and to eventually initiate a positive feedback that leads to recovery. The failed or delayed recovery of the stocks highlights furthermore the importance to detect discontinuous dynamics in advance, in order to avoid unpleasant surprises [1,64,74].

Climate change will continue to cause a considerably warmer Atlantic Ocean [75] and our results show that increasing SST will have negative repercussions for most of the Atlantic cod stocks that already live at their upper thermal tolerance limits [66,76]. Indeed, the increase in temperature in these areas will strongly reduce the productivity of most of the cod stocks as shown in other studies [51,66]. However, the few stocks residing at or close to their lower thermal tolerance limits are expected to benefit from warming (figure 3*i–h*). Warming and reduced fishing pressure initiated the recovery of the Icelandic cod stock, which could be even more pronounced if fishing mortality would be reduced to below *F*_MSY_. To date, northeast Arctic cod is benefiting the most from ocean warming [77]. According to our cusp model, the northeast Arctic cod population resides in (or is on the verge of) a high SB stable state (recent high SB values are outside or at the tip of the bifurcation area; figure 3*i*), a development supported by effective management [78].

Our results demonstrate how ocean warming induces a nonlinear and discontinuous relationship between stock size and fishing pressure in most of the Atlantic cod stocks. These catastrophic dynamics are fundamental to understand because they can lead to management failure and unpleasant ecological surprises [64,79]. Even though recorded in many areas and at different ecosystem levels, catastrophic dynamics are still largely ignored in fisheries and ecosystem management because of the challenges of analysing them and the few models available [15,80,81]. We show that application of catastrophe theory can help to better understand discontinuous dynamics, but also the interaction of external drivers, resilience and proximity to tipping points.

Our study has limitations resulting in particular from uncertainties in the stock assessment data used. Also, the methodological confines of the stochastic cusp modelling approach, such as deficiencies in accounting for autocorrelation in time series [49] and known uncertainties in model comparison using indices such as R^2^ [48] and AIC [82] might influence the validity of our results. While improving the methodology is beyond the scope of our study, we conducted a careful approach to validate our modelling results using multiple criteria as suggested and applied in other studies [48,82,83]. The results of this multifaceted validation approach give us confidence that the stochastic cusp model is a valid model to describe the dynamics of Atlantic cod stocks and is frequently superior to the more simpler linear and logistic models.

Nevertheless, we acknowledge that a theoretical model such as the stochastic cusp model cannot reveal the underlying ecological processes which can only be proven applying an experimental approach. Unfortunately, large natural populations such as Atlantic cod are impossible to manipulate experimentally. Hence, we acknowledge that a detailed understanding of the effect of temperature on biological processes such as growth [84,85] and recruitment [86,87] or ecosystem changes such as trophic cascades is needed to better explain our statistical model results and should be performed at the stock level. Nonetheless, we believe our modelling results provide advances in explaining the interacting effects of the two drivers in identifying catastrophic dynamics of Atlantic cod stocks and their possible recovery potential.

In conclusion, we here contribute a novel assessment of the vulnerability of Atlantic cod stocks to climate change, explicitly accounting for the potential of nonlinear and state-dependent dynamics that will be useful for ecosystem-based management of the oceans. Other resource species may follow similar catastrophic dynamics as we have here demonstrated for Atlantic cod, and as such we suggest that a precautionary approach accounting for environmental change is warranted for the sustainable management of living resources under the expected future climate change [15,58,81]. Finally, we demonstrated the usefulness of the stochastic cusp modelling approach to explain abrupt changes in ecological systems, which hopefully will spur application as seen in other scientific disciplines.

## Supplementary Material

supplementary information

## Data accessibility

Data available from the Dryad Digital Repository: https://doi.org/10.5061/dryad.037jr6v [88].
