# Supplementary material for: Catastrophic dynamics limit Atlantic cod recovery
Source: Proc Biol Sci. 2019 Mar 13;286(1898):20182877. doi: 10.1098/rspb.2018.2877 (PMC6458326; doi:10.1098/rspb.2018.2877)
Supplement: supplementary information [file rspb20182877supp1.pdf]

Supplementary Information for

Catastrophic dynamics limit Atlantic cod recovery

Camilla Sguotti[a], Saskia A. Otto[a], Romain Frelat[a], Tom J. Langbehn[b], Marie Plambech Ryberg[c], Martin Lindegren[c], Joël M. Durant[d], Nils Chr. Stenseth[d], Christian Möllmann[a]

[a]Institute for Marine Ecosystem and Fisheries Science (IMF), Center for Earth System Research and Sustainability (CEN), University of Hamburg, Große Elbstraße 133, 22767 Hamburg, Germany.

[b]Department of Biological Sciences, University of Bergen, Thormøhlensgate 53 A & B, 5006 Bergen, Norway

[c]National Institute of Aquatic Resources, Technical University of Denmark (DTU Aqua), Kemitorvet, Building 202, DK-2800 Kgs. Lyngby, Denmark.

[d]Centre for Ecological and Evolutionary Synthesis (CEES), University of Oslo, Dept. of Biosciences, P.O. Box 1066 Blindern, NO-0316 Oslo, Norway

Camilla Sguotti
Email: camilla.sguotti@uni-hamburg.de

This PDF file includes:

Supplementary text
Figs. S1 to S5
Tables S1 to S6

Supplementary Information Text

Recovery analyses

Atlantic cod stocks have shown synchronous collapses and very little recovery in many areas of the North Atlantic. In order to confirm these results and back up our analyses on the non-linearity of cod stocks behaviour, we performed analyses on collapse and recovery. Collapse was investigated using statistical change point and trend analyses on long-term time-series of Spawner Biomass (SB). We applied *Bayesian Change Point Analysis* (bcp(1)), which returns a posterior probability of a change to occur at each year of the time series. In order to identify a breakpoint in time indicating a major shift, we validated the bcp results with a trend analysis. The trend analysis estimates the second derivative of the SB time-series smoothed by fitting a Generalized Additive Model (GAM) to time (2) and allow to detect years with increases in the rate of change in the SB time-series. Combining the two approaches allowed us to identify for each cod stock the year in which a major change point occurred and to better understand the trends in SB (Fig.1). A high frequency of collapses occurred during the early 1990s with 15 of the stocks declining to below 50% of pre-collapse SB. Only North-East Arctic cod collapsed already before the 1960s and afterwards SB increased abruptly, while the stocks in the Celtic Sea, on the Faroe Plateau and in the Gulf of Maine exhibited oscillating trajectories and a very recent stock depletion.

In order to compare biomass trajectories across cod stocks we used Principal Component Analysis (PCA) using SB data from 1983 to 2016. Missing values at the end of the time-series (i.e. when the time-series did not include 2016 or before) were substituted with the last value to allow for a PCA with all stocks. The main mode of variability across stocks (PC1, 58%) shows constantly declining SB since the early 1990s (Fig. S1a) to which 16 of the 19 cod stocks were positively correlated (Fig. S1b). A second mode (PC2, 20%) indicates partial recovery since the mid-2000s, a temporal pattern highly positively correlated to the North-East Arctic, Icelandic and Flemish Cap, as well as North Sea cod (Fig. S1c).

Finally, we calculated a *Recovery Index* (RI) for all Atlantic cod stocks by comparing the average of the SB over the last 5 years (SB_{mean}) to the pre-collapse SB ($SB_{pre\text{-}collapse}$):

$$RI = \ (SB_{mean}/SB_{pre-collapse}) \times 100$$

Afterwords we classified all cod stocks into three recovery classes: (i) *collapsed* – RI ≤ 20%, (ii) *recovering* – RI >20 and ≤50%, and (iii) *recovered* – R >50%. We found only two stocks *recovered*, i.e. North-East Arctic and Flemish Cap cod, and six stocks *recovering*. 11 of the 19 stocks can still be considered *collapsed* (Fig. S2).

1. Erdman C, Emerson JW (2007) bcp: an R package for performing a Bayesian analysis of change point problems. *J Stat Softw* 23(3):1–13.
2. Fewster RM, Buckland ST, Siriwardena GM, Baillie SR, Wilson JD (2000) Analysis of Population Trends for Farmland Birds Using Generalized Additive Models. *Ecology* 81(7):1970–1984.

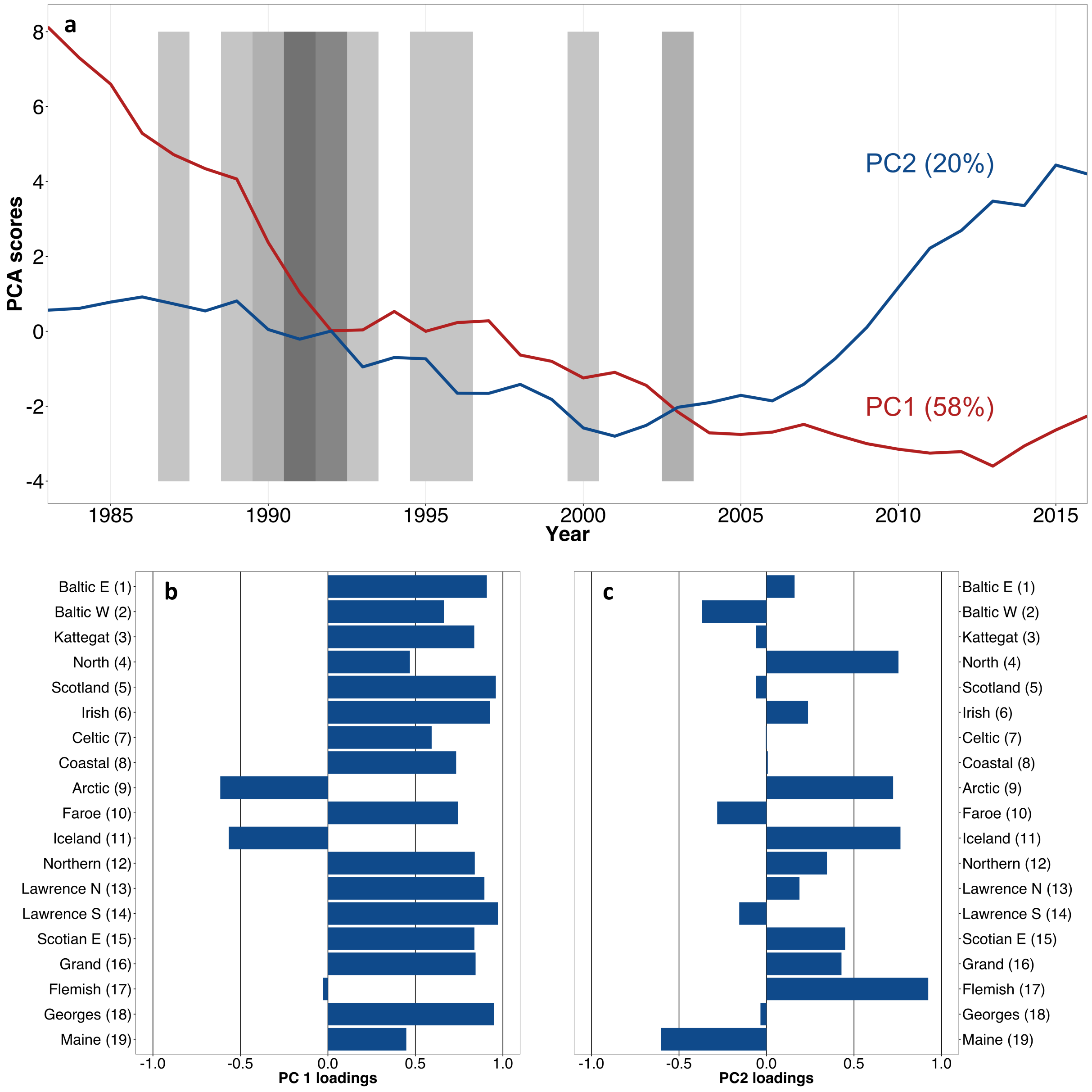

Figure S1. Collapse and recovery modes.

a, Dominant modes of Atlantic cod stock collapse and recovery revealed by PCA. PC1 (red line) indicates that most of the stocks are still in a *collapsed* state, PC2 (blue line) indicates a *recovery* pattern. Grey vertical bars indicate the number of stocks showing a significant change in any given year (light grey=1, dark grey=4). **b**, Loadings of Atlantic cod stocks on PC1 indicating that most of the stock´s trajectory have a high correlation to the *collapsed* mode. **c**, Loadings of Atlantic cod stocks on PC2 indicating the *recovery* state of cod stocks, i.e. their correlation to the recovery mode indicated by PC2. Stock names and numbers according to Table S1.

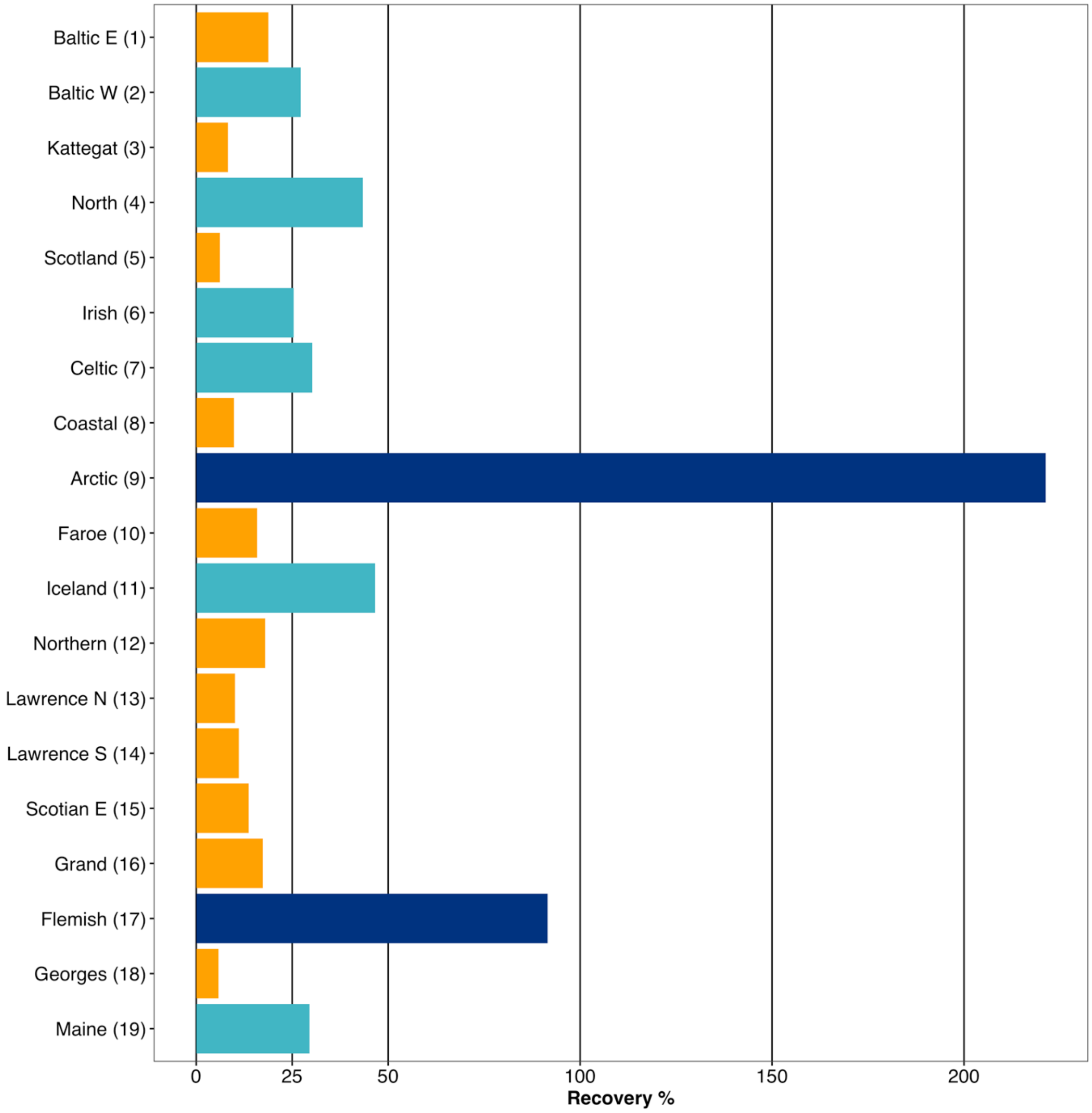

Figure S2. Recovery index (RI).
Recovery state of Atlantic cod stocks indicated by a recovery index (SI methods) indicating stocks to be still *collapsed* (orange), *recovering* (light blue) and *recovered* (dark blue). Stock names and numbers according to Table S1.

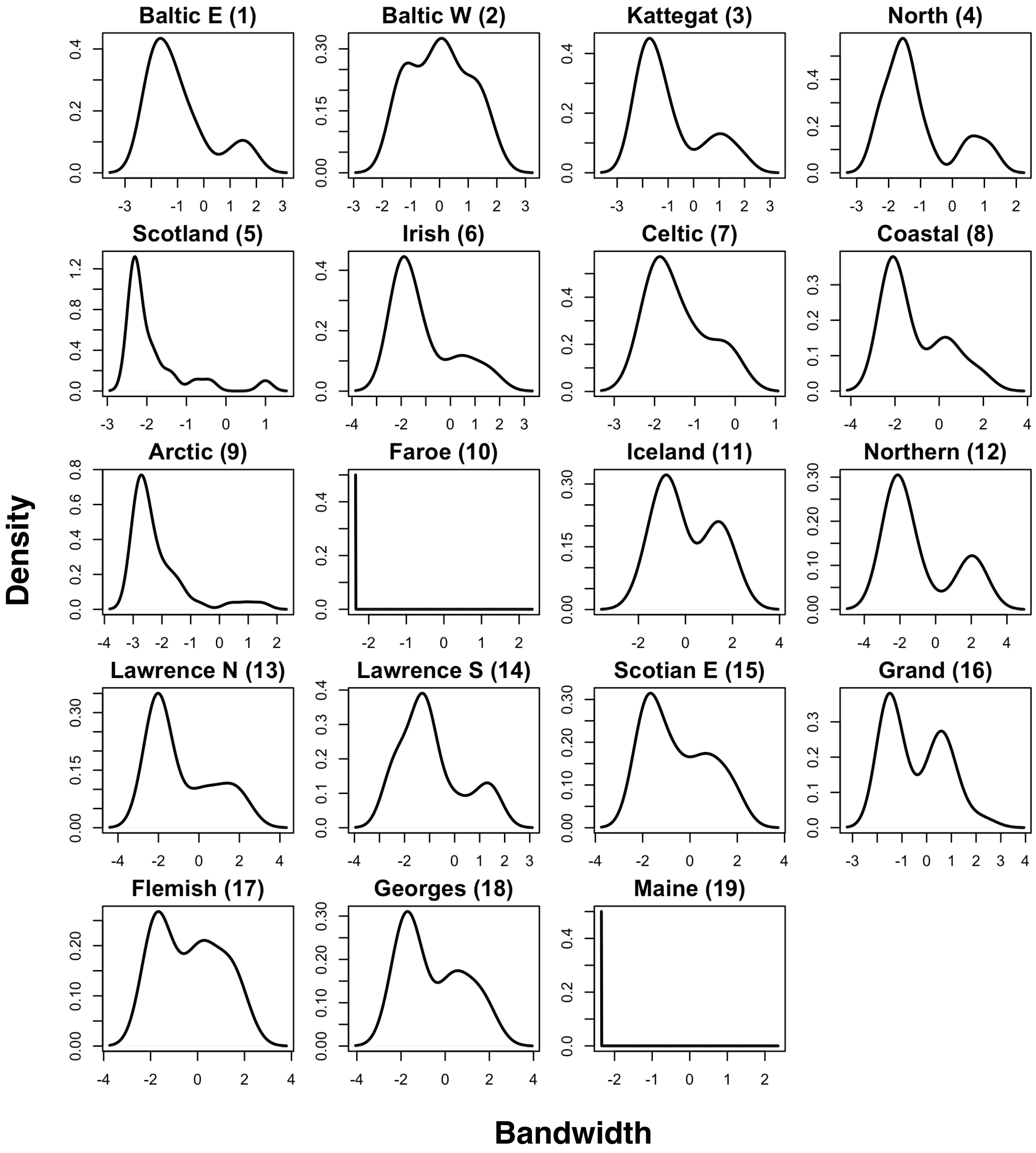

Figure S3. Bimodality in the bifurcation set.
Evaluation of bimodality inside the bifurcation set (upper left inlet) as an indicator of validity of stochastic cusp models for Atlantic cod stocks. Empty plots indicate models with only one point in the bifurcation set. Stock names and numbers according to Table S1.

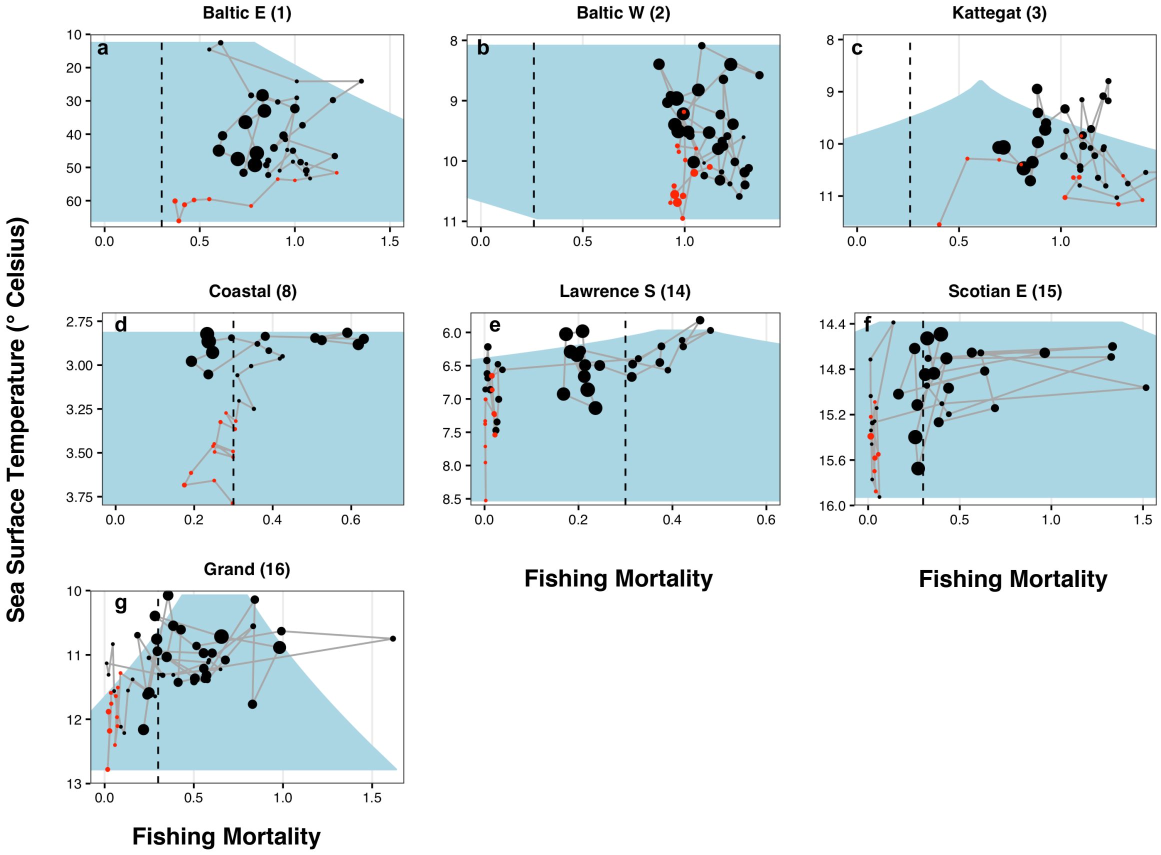

Figure S4. 2D representation of cusp model results.
Stock trajectories of Atlantic cod stocks depending on fishing mortality (F_M) and sea surface temperature (SST, except for Baltic E which is modelled depending on the extend of anoxic areas; see method). Dot size is scaled according to the annual spawners biomass (SB) of each stock. Red dots represent years $\geq$2004. The blue area indicates the *bifurcation set* of the *cusp model*. Vertical dotted lines indicate the present management target specific for each stock (Table S3) Stock names and numbers according to Table S1.

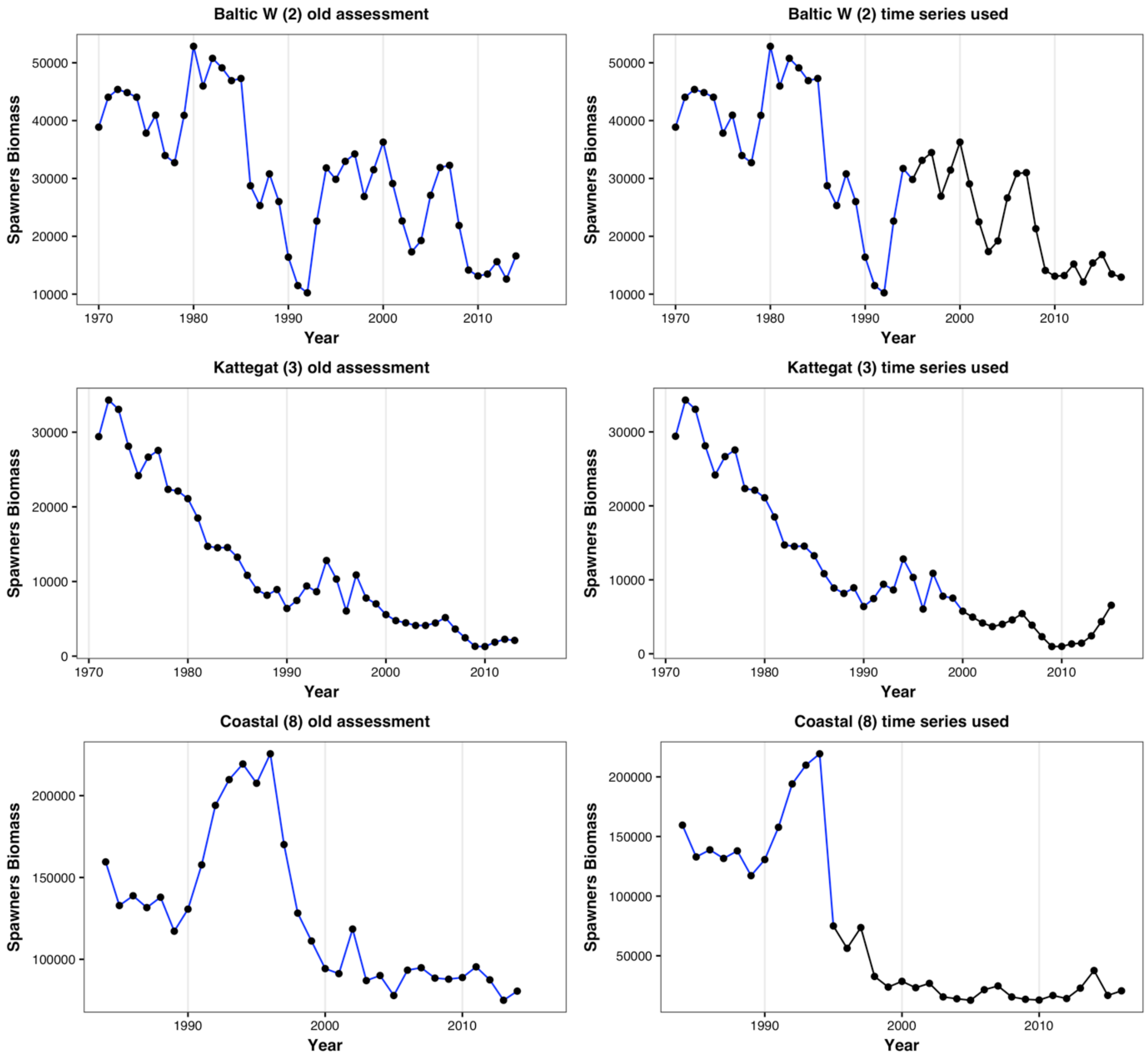

Figure S5. Stock assessments series used for three short time series stocks.
On the left the time series of SB of the older assessments, 2014, 2015 and 2016 for respectively Western Baltic, Kattegat and Coastal Cod. On the right the combined time series of the old stock assessments (in blue) and of the new 2017 stock assessments (black).

Table S1. Stock assessment data of Atlantic cod stocks.

No.	Stock name	Abbreviation	Management area	Stock assessment models*	Institution/ group	Contact person or other data source
1	Eastern Baltic[1]	Baltic E	25-32	SAM	ICES/ WGBAFS	Margit Eero
2	Western Baltic[2]	Baltic W	22-24	SAM	ICES/ WGBAFS	Margit Eero
3	Kattegat[1,2]	Kattegat	IIIa/21	SAM	ICES/ WGBAFS	Johan Lövgren
4	North Sea	North	IV – VIId – IIIa	SAM	ICES/ WGNSSK	Alexander Kempf
5	West of Scotland	Scotland	VIa	TSA	ICES/ WGCSE	Rui Catarino
6	Irish Sea	Irish	VIIa	SAM	ICES/ WGCSE	Colm Lordan
7	Celtic Sea	Celtic	VIIe – VIIk	XSA	ICES/ WGCSE	Colm Lordan
8	Coastal Barents Sea[2]	Coastal	I – I	Survey SSB and F from VPA	ICES/ AFWG	Gjert Endre Dingsor
9	Northeast Arctic	Arctic	I – II	XSA	ICES/ AFWG	Gjert Endre Dingsor
10	Faroe Plateau	Faroe	Vb1	XSA	ICES/ NWWG	Petur Steingrund
11	Iceland	Iceland	Va	Forward based statistical catch at age model	ICES/ NWWG	Einar Hjorleifsson
12	Northern cod	Northern	2J3Kl	State space model	DFO	DFO(2016)[3]
13	Northern Gulf of st. Lawrence	Lawrence N	3Pn4Rs	SPA	DFO	Claude Brassard
14	Southern Gulf of st. Lawrence	Lawrence S	4T4Vn	SCA	DFO	Doug Swain
15	Eastern Scotian Shelf	Scotian E	4VsW	VPA	DFO	Swain & Mohn (2012)[4]
16	Grand Banks	Grand	3NO	SPA	NAFO	NAFO (2015)[5]
17	Flemish Cap	Flemish	3M	Bayesian model	NAFO	Diana Gonzalez
18	Georges Bank	Georges	5z	Age structured model	NOAA	Loretta O´Brien
19	Gulf of Maine	Maine	5y	Age structured model	NOAA	Mike Palmer

Stock number (No.), stock name, abbreviation, management area code, stock assessment models used and the institution (or working group within the institution) conducting the stock assessment. Scientists providing data (or other data sources)

* SAM – State-space assessment model, TSA – Analytical age-based assessment (time series analysis), XSA – Extended survivor analysis, SPA – Sequential population analysis, SCA – Statistical catch at age, VPA – Virtual population analysis

[1] Stocks that in the last year failed the assessment

[2]Stocks where, in order to have longer time series, we mixed old assessments and new ones, after checking for same trends and stock dimensions.

[3]DFO (2016) Stock Assessment of Northern Cod (NAFO Divs. 2J3KL) in 2016. DFO Can. Sci. Advis. Sec. Sci. Advis. Rep. 2016/026

[4] Swain DP, Mohn RK (2012) Forage fish and the factors governing recovery of Atlantic cod (Gadus morhua) on the eastern Scotian Shelf. Can. J. Fish. Aquat. Sci. 69: 997–1001.

[5] NAFO (2015) Assessment of the Cod Stock in NAFO Divisions 3NO. NAFO SCR Doc. No. 15/034.

Table S2. Summary of data used in the analysis.

No.	SB (t)	FM	SST (°C)	ΔT(°C)	Assessment period
1	62193 – 643064	0.37 – 1.35	6.9 – 9.7	13.5 – 18.9	1966-2013
2	10229 – 52839	0.874 – 1.36	8.0 – 10.9	13. 4 – 19.0	1970-2016
3	977 – 34303	0.4 – 1.52	8.8 – 11.5	11.0 – 16.4	1971-2014
4	43739 – 274855	0.35 – 1.07	9.3 – 11.7	8.9 – 13.2	1963-2016
5	1435 – 40438	0.66 – 1.18	10.0 – 11.4	4.2 – 6.0	1981-2016
6	1389 – 19791	0.76 – 1.38	10.4 – 12.0	6.6 – 9.3	1968-2016
7	3397 – 26324	0.35 – 0.99	12.0 – 13.7	5.6 – 8.5	1971-2016
8	12709 – 219345	0.17 – 0.63	2.8 – 3.8	3.8 – 4.5	1984-2016
9	102610 – 2692927	0.21 – 1.02	2.5 – 3.8	3.7 – 4.8	1946-2016
10	16786 – 123077	0.19 – 0.82	8.9 – 10.3	2.9 – 4.9	1959 -2016
11	121063 – 936957	0.27 – 0.89	5.6 – 7.5	3.3 – 5.7	1955-2016
12	9680 – 940750	0.01 – 0.22	4.6 – 7.2	8.8 – 11.6	1983-2015
13	6774 – 200271	0.03 – 1.96	4.6 – 7.2	14.1 – 17. 6	1974-2015
14	33714 – 348193	0.01 – 0.48	5.8 – 8.5	15.5 – 18.7	1971-2014
15	4412 – 155525	0.01 – 1.52	14.3 – 16.0	11. 4 – 14.0	1970-2010
16	4231 – 125043	0.01 – 1.61	10.0 – 12.7	9.5 – 14.6	1959-2015
17	1697 – 42514	0.003 – 1.52	13.6 – 16.0	6.5 – 9.9	1972-2015
18	4066 – 98527	0.4 – 1.33	13.0 – 15.5	12.8 – 15.6	1978-2014
19	2526 – 21939	0.34 – 1.53	9.0 – 11.7	13.0 – 15.9	1982-2014

Ranges of spawner biomass (SB), fishing mortality (FM), sea surface temperature (SST) and its annual variability (ΔSST) for the respective assessment period. Stock numbers (No.) according to Table S1.

Table S3. Management reference points for Atlantic cod stocks

No.	FM reference point	FM reference point value
1	-	-
2	F_{MSY}	0.26
3	-	-
4	F_{MSY}	0.33
5	F_{MSY}	0.17
6	F_{MSY}	0.3
7	F_{MSY}	0.35
8	-	-
9	F_{MSY}	0.4
10	F_{MSY}	0.32
11	HR_{MSY}	0.2
12	*	*
13	*	*
14	*	*
15	*	*
16	F_{lim}	0.3
17	F_{lim}	0.13
18	F_{MSY}	0.17
19	F_{MSY}	0.18

fishing mortality (FM) management reference points and their values. “-“ shows stocks without available stock assessments. “*” indicates stocks for which FM reference points are not given in reports; Stock numbers (No.) according to Table S1.

Table S4| Cusp model evaluation.

No.	Percentage in bifurcation set	Bimodality
**1**	97.9	(+)
**2**	100	(+)
**3**	84	(+)
**4**	44	(+)
**5**	61	(+)
**6**	59.1	(+)
**7**	10	(-)
**8**	100	(+)
**9**	60	(-)
**10**	1.7	(-)
**11**	20.9	(+)
**12**	100	(+)
**13**	100	(+)
**14**	88.6	(+)
**15**	100	(+)
**16**	82.1	(+)
**17**	76.7	(+)
**18**	86.1	(+)
**19**	3	(-)

Two criteria for a *cusp model* to be considered valid; (i) percentage of data points inside the bifurcation area (should be > 10%) and bimodality of the state variable inside the bifurcation area. Models underlined in grey are not valid cusp models according to these criteria. Stock numbers (No.) according to Table S1.

Table S5. Cusp model results for the invalid models.

Stock	α_0	α_1	β_0	β_1	w_0	w_1	R^2	ΔAIC
**Celtic (7)**	-4.18(1.58)**	3.65(1.73)*	-50.38(17)**	3.87(1.26)***	-2.64(0.29)***	1.71E-04(1.91E-05)***	0.0064	784
**Faroe (10)**	-2.8(1.75)	1.90(1.87)	-34.42(7.14)***	3.60(0.75)***	-2.31(0.13)***	2.39E-05(5.35E-06)***	0.3	1184
**Maine (19)**	-0.12(1.0)	-1.19(1.03)	--27.80(8.88)**	2.63(0.84)**	-2.28(0.41)***	1.51E-04(3.48E-05)***	0.25	556

Results of the invalid (see Table S4) *cusp models* for Atlantic cod stocks (stock numbers according to Table S1 are indicated in brackets). Reported are estimated model parameters (with standard errors) α_0/α_1 (for fishing mortality - F_M), β_0,/β_1 (for sea surface temperature -SST) and for w_0,/w_1 (spawner biomass - SB, as the state variable), Stars indicate the significance level of the estimated parameters (* <0.05, ** <0.005, *** <0.0005). Furthermore, the R^2 (Cobb´s Pseudo R^2) indicates the quality of the cusp model fit and the ΔAIC_c (difference between the AIC_c of the cusp and logistic model) is given for comparison of the cusp and the alternative models.

Table S6. Results of the lagged models

Stock	α_0	α_1	β_0	β_1	w_0	w_1	R^2	ΔAIC
**Baltic W (2)**	-5.49(9.76)	0.49(3.06)	-21.57(11.64)	1.94(0.90)*	-2.18(0.48)***	0	0.25	863
**Kattegat (3)**	0.69(0.17)***	-1.22()	-18.29(4.27)***	2.03(3.97)***	-2.49(0.096)***	1.33E-04(7.5E-06)***	0.78	816
**North (4)**	0.71(0.5)	-1.9(0.80)*	-18.41(5.25)***	1.92(0.49)***	-3.04(0.23)***	1.62E-05(1.7E-06)***	0.41	1187
**Scotland (5)**	2.41(2.12)	-4.04(2.79)	-64.35(11.13)***	6.22(1.05)***	-2.67(0.199)***	9.77E-05(1.2E-05)***	0.65	660
**Irish (6)**	-0.31(0.44)	-0.59(0.49)	-45.87(9.77)***	4.18(0.84)***	-2.30(0.18)***	1.67E-04(2.75E-05)***	0.44	840
**Celtic (7)**	-4.8(2.18)*	3.63(2.14)	-31.07(11.71)**	2.45(0.86)**	-2.69(0.25)***	1.38E-04(2.38E-05)***	0.15	882
**Coastal (8)**	-0.42(0.45)	-0.06(1.38)	-11.44(3.52)**	4.83(1.19)***	2.64(0.18)***	1.38E-04(1E-06)***	0.84	762
**Arctic (9)**	2.52(0.26)***	-8.92()	21.91(0.49)***	-5.70()	-3.22(0.11)***	1.85E-06(1.07E-07)***	0.9	1869
**Faroe (10)**	-1.56(1.05)	0.42(1.45)	41.7(7.29)***	4.45(4.87E-06)***	-2.56(0.75)***	2.83E-05(0.206)	0.4	1149
**Iceland (11)**	3.43(1.04)**	-11.2(3.24)***	17.99(4.23)***	-2.39(4.11E-07)***	-3.41(0.61)***	6.19E-06(0.1677)	0.81	1480
**Northern (12)**	-2.08(0.88)*	11.18(4.59)*	11.11(2.98)***	-1.23(0.15)***	-2.46(0.514)***	5.29E-06(3.3E-07)***	0.93	749
**Lawrence N (13)**	-0.33(0.17)*	0.12(0.27)	-3.038(2.8)	1.30(0.53)*	-2.63(0.15)***	2.54E-05(1.4E-06)***	0.91	901
**Lawrence S (14)**	-0.66(0.23)**	2.01(0.99)*	-13.01(3.43)***	2.29(0.52)***	-2.92(0.20)***	1.35E-05(1.02E-06)***	0.8	993
**Scotian E (15)**	-0.50(0.03)***	0.64()	-22.97(9.48)*	1.68(0.63)**	-2.15(0.16)***	2.7E-05(1.9E-06)***	0.80	866
**Grand (16)**	-0.9(0.28)**	1.41(0.50)**	-1.46(5.16)	0.27(0.46)	-1.87(0.15)***	3.48E-05(2.88E-06)***	0.74	1139
**Flemish (17)**	-0.48(0.25)	0.57(0.38)	-20.8(8.60)*	1.49(0.57)**	-1.99(0.20)***	1.13E-04(1.075E-05)***	0.68	769
**Georges (18)**	2.05(0.86)*	-3.62(1.36)**	-13(9.04)	1.07(0.64)	-2.259(0.17)***	4.24E-05(3.918E-06)***	0.71	734
**Maine (19)**	0.4(1.11)	-1.56(1.13)	-30.91(10.28)**	2.87(0.92)**	-2.10(0.49)***	1.5E-04(4.07E-05)	0.31	534

Results *cusp models* for Atlantic cod stocks (stock numbers according to Table S1 are indicated in brackets) using lagged sea surface temperature (SST). Baltic E (1) is not shown since SST was not used in its model. Reported are estimated model parameters (with standard errors, where not indicated the model did not converge) α_0/α_1 (for fishing mortality - F_M), $\beta_0,/\beta_1$ (for SST lagged at the Year of recruitment) and for $w_{0,}/w_1$ (spawner biomass - SB, as the state variable). Stars indicate the significance level of the estimated parameters (* <0.05, ** <0.005, *** <0.0005).

Furthermore, the R^2 (Cobb´s Pseudo R^2) indicates the quality of the cusp model fit and the ΔAIC_c (difference between the AIC_c of the cusp and logistic model) is given for comparison of the cusp and the alternative models.
